# Competitive cation binding computations of proton balance for reactions of the phosphagen and glycolytic energy systems within skeletal muscle

**DOI:** 10.1371/journal.pone.0189822

**Published:** 2017-12-21

**Authors:** Robert Andrew Robergs

**Affiliations:** School of Exercise and Nutrition Sciences, Faculty of Health, Queensland University of Technology, Kelvin Grove, Queensland, Australia; Heidelberg University, GERMANY

## Abstract

Limited research and data has been published for the H^+^ coefficients for the metabolites and reactions involved in non-mitochondrial energy metabolism. The purpose of this investigation was to compute the fractional binding of H^+^, K^+^, Na^+^ and Mg^2+^ to 21 metabolites of skeletal muscle non-mitochondrial energy metabolism, resulting in 104 different metabolite-cation complexes. Fractional binding of H^+^ to these metabolite-cation complexes were applied to 17 reactions of skeletal muscle non-mitochondrial energy metabolism, and 8 conditions of the glycolytic pathway based on the source of substrate (glycogen vs. glucose), completeness of glycolytic flux, and the end-point of pyruvate vs. lactate. For pH conditions of 6.0 and 7.0, respectively, H^+^ coefficients (-‘ve values = H^+^ release) for the creatine kinase, adenylate kinase, AMP deaminase and ATPase reactions were 0.8 and 0.97, -0.13 and -0.02, 1.2 and 1.09, and -0.01 and -0.66, respectively. The glycolytic pathway is net H^+^ releasing, regardless of lactate production, which consumes 1 H^+^. For glycolysis fueled by glycogen and ending in either pyruvate or lactate, H^+^ coefficients for pH 6.0 and 7.0 were -3.97 and -2.01 (pyruvate), and -1.96 and -0.01 (lactate), respectively. When starting with glucose, the same conditions result in H^+^ coefficients of -3.98 and -2.67, and -1.97 and –0.67, respectively. The most H^+^ releasing reaction of glycolysis is the glyceraldehyde-3-phosphate dehydrogenase reaction, with H^+^ coefficients for pH 6.0 and 7.0 of -1.58 and -0.76, respectively. Incomplete flux of substrate through glycolysis would increase net H^+^ release due to the absence of the pyruvate kinase and lactate dehydrogenase reactions, which collectively result in H^+^ coefficients for pH 6.0 and 7.0 of 1.35 and 1.88, respectively. The data presented provide an extensive reference source for academics and researchers to accurately profile the balance of protons for all metabolites and reactions of non-mitochondrial energy metabolism, and reveal the greater role of glycolysis in net H^+^ release than previously assumed. The data can also be used to improve the understanding of the cause of metabolic acidosis, and reveal mechanistic connections between H^+^ release within and from muscle and the electrochemical neutrality concepts that further refine acid-base balance in biological solutions.

## Introduction

Reading of the research and educational literature on the proton (H^+^) balance of cytosolic (non-mitochondrial) reactions of energy metabolism within skeletal muscle reveals inconsistencies in interpretations of the stoichiometry, source and consumption of H^+^. For example, despite the prior work by Kushmerick et al. [1] and Vinnakota et al. [2] on the competitive binding of multiple cations to metabolites within skeletal muscle, as well as Li et al. [3] on the cation dependency of thermodynamic properties of multiple metabolic pathways, numerous research studies [4–12] have been interpreted with a more simplistic understanding of H^+^ balance. For example, and as presented in Eqs 1–4, the reactions of non-mitochondrial energy metabolism are presented with summary reactions revealing the consumption of 1 H^+^ in the creatine kinase reaction, the release of 2 H^+^ from glycolysis, and the consumption of 2 H^+^ by lactate production. Such summary reactions lead to the interpretation that lactate production makes glycolysis H^+^ neutral (Eq 4).

$$\mathrm{CrP}+\mathrm{ADP}+H^{+}\overset{CK}{↔}ATP+Cr$$ (1)

$$Glucose+2\;ADP+2\;Pi+2\;NAD^{+}\overset{glycolysis}{↔}2\;Pyruvate+2\;ATP+2\;NADH+2\;H_{2}O+2\;H^{+}$$ (2)

$$2\;Pyruvate+2\;NADH+2\;H^{+}\overset{LDH}{↔}2\;Lactate+2\;NAD^{+}$$ (3)

$$Glucose+2\;ADP+2\;Pi\;\overset{glycolysis+LDH}{↔}\;2\;Lactate+2\;ATP+2\;NAD^{+}$$ (4)

While such simplified expressions of chemical reactions are conceptually easy to comprehend, the reality of the biochemistry of proton balance in muscle energy metabolism is far more complex. All the metabolites of the phosphagen and glycolytic energy systems have one or multiple sites for ionization. In addition, each ionizable site is susceptible to not only H^+^ binding, but also binding by other cations present in meaningful concentrations, such K^+^, Mg^2+^ and Na^+^. Consequently, when quantifying the balance of H^+^ binding to metabolites, or H^+^ consumption or release during chemical reactions, it is important to account for the competitive binding of multiple cations.

In prior work, the present author has argued and presented a wealth of evidence as to the falsehood of the concept of a lactic acidosis, and the validity for interpreting metabolic acidosis to be caused by an imbalance in proton release and consumption during muscle energy metabolism [9–12]. While the focus of this past work was not to provide accurate computations of H^+^ balance in skeletal muscle energy metabolism, a fair criticism was the incomplete presentation and computations of the actual H^+^ balance of the reactions at question [5,7,8]. Due to such criticisms, work was undertaken to research the H^+^ balance for all reactions of non-mitochondrial skeletal muscle energy metabolism.

Limited prior work has been done to document the proportion of bound and unbound metabolite-cation complexes of intermediary metabolism in skeletal muscle, and consequently, to accurately reveal the extent of H^+^ binding to these metabolites. Kushmerick [1] computed the binding coefficients of H^+^, Mg^2+^ and K^+^ to adenosine triphosphate (ATP), adenosine diphosphate (ADP), inorganic phosphate (Pi), creatine phosphate (CrP), creatine (Cr) and lactate (La). However, the glycolytic pathway, the reactions of glycogenolysis, and additional reactions of the phosphagen energy system (adenylate kinase and AMP deaminase reactions), which can all influence H^+^ balance, were not included in this compilation.

Vinnakota et al. [2] performed an extensive computation of metabolite-H^+^ binding in their work of modeling the energetics of intermediary metabolism. These authors included reactions of glycolysis and glycogenolysis in their model, and corrected all reference metabolite-cation binding constants to an ionic strength of 0.1 M, and to temperatures ranging from 25 to 37°C. Nevertheless, as with Kushmerick et al. [1], not all reactions were included from the phosphagen energy system, and data for fractional H^+^ binding were not reported for all competing cation-metabolite interactions or all reactions of glycogenolysis and glycolysis.

Li et al. [3] compiled a database of thermodynamic properties for the reactions of glycolysis, the TCA cycle and pentose phosphate pathway. While these authors adjusted their data for competing cations, their purpose was not to report on H^+^ implications and therefore no data were presented on the pH dependent H^+^ exchange for these reactions. In addition, the LDH reaction was not included in their work.

The incomplete prior research of the stoichiometry of proton balance for all reactions of skeletal muscle non-mitochondrial energy metabolism prevent accurate computations of the proton release and metabolic buffering accompanying muscle contraction. The purpose of this research was to list the binding constants of all the metabolites of non-mitochondrial metabolism for all competing cations. The binding constants were then used to compute the fractions of bound and unbound metabolite-cation complexes across a pH range from 6.0 to 7.0. These fractions were used to compute the H^+^ coefficients for all reactions of the phosphagen and glycolytic energy systems in skeletal muscle across this pH range. The pH specific H^+^ coefficients for the reactions of non-mitochondrial intermediary metabolism resulting from this research should improve the understanding of proton balance in contracting skeletal muscle and the biochemistry of metabolic acidosis. Such understanding has benefit to the physiological interpretation of the metabolic stress of repeated intense muscle contractions in both education and research settings, and for other systemic causes of metabolic acidosis such as resulting from cardiovascular, pulmonary, or metabolic diseases.

## Materials and methods

### Understanding the ionization equilibrium and dissociation constant

For conceptual completeness, it is important to understand the analytical basis for the binding constant of a molecule and interacting cation. For a given ligand molecule (L) and binding metallic cation (M^n+^), inherent properties of attraction between the two species will result in a ratio between bound and unbound components, referred to as the ionization equilibrium, binding constant, association constant or dissociation constant (Eqs 5 and 6). However, this terminology is often used incorrectly, as association and dissociation constants are not synonymous; the dissociation constant is the reciprocal of the association constant. For computations of H^+^ exchange in chemical reactions, we are concerned with the dissociation constant (K_M+_). The K_M+_ is a measure of the extent of a reversible dissociation between two molecular species. When M^n+^ is a H^+^, K_H+_ is referred to as the acid dissociation constant (K_a_).

KM+=[L][M][LM] (5)

For metabolite-cation complexes involving H^+^ under standard pH conditions (pH = 7.0), the denominator is small, and hence K_M+_ is relatively large. Also, the greater the bound species compared to the unbound species, the larger the K _M+_. For example, the K_a_ for lactic acid, lactate and H^+^ is expressed in Eq 6.

Ka=[lactate][H+][lacticacid] (6)

By convention, K_a_ is expressed to the log10 (pK). For example, the metabolite-cation complex of lactate-H^+^ (lactic acid) has a pK_a_ = 3.67, and computational expressions of the K_a_ and pK_a_ of lactic acid are shown in Eqs 7–10.

logKa=pKa=log([lactate][H+][lacticacid]) (7)

pKa[lactate][H+][lacticacid]=3.67 (8)

$$3.67=\log\;K_{a}$$ (9)

$$K_{a}=10^{3.67}=4677.35514$$ (10)

In biological solutions, the extent of binding between L and M^n+^ is dependent on 1) inherent properties of attraction between two species quantified by the pK, 2) the concentrations of L ([L]) and M ([M^n+^]), 3) the concentration(s) of any other cation(s) that can bind to L, and 4) the pK_M+_ values of the L and all M^n+^ binding. Consequently, for conditions where [M^n+^] is relatively high, a low pK_M+_ may still coincide with high M^n+^ binding. In addition, when more than one M^n+^ competes for L binding, the actual stoichiometry of binding for a specific M^n+^ will be less than that computed singularly. This fact renders computations of the balance of H^+^ for specific reactions solely from the pK_a_ of the metabolite to be erroneous. The magnitude of the error in computing singular H^+^ binding increases with increasing competition between multiple M^n+^, especially when the sum of K and [M^n+^] are large, as has been explained by Kushmerick et al. [1]. For valid data of L-M^n+^ binding, multiple competing cations need to be included in computations.

### Data reference sources

The most recent electronic version of the National Institute of Standards and Technology (NIST) Standard Reference Data Base [13] was purchased to obtain the latest adjustments to the critical stability constants, first published by Robert Smith and Arthur Martell in 1972. The molecules of Table 1 were identified in this reference resource, and the dissociation constants (pK) and enthalpy of dissociation (ΔH) at 25°C and 0.1 M ionic strength, or the closest conditions to these, were recorded for each metabolite and cation complex with each and any combination of H^+^, Mg^2+^, K^+^ and Na^+^. Metabolite-cation interactions not included in the NIST data base were obtained from original research as identified by Kushmerick et al. [1] and Vinnakota et al. [2], and are presented in Table 1.

**Table 1:** The association constant (pK) and enthalpy of dissociation (ΔH) of select metabolite and cation complexes of muscle energy catabolism for reference conditions (temperature °C;ionic strength M).

Metabolite-Cation Complexes		Reference Data			pK (25, 0.1)
Generic Name	[] ^#^	Binding	pK	ΔH	
***Phosphagen System***					
Hydrogen phosphate	4.0	Pi-H	11.59(25;0.1)	-15.0(25;0.1)	11.59
		Pi-H_2_	6.75(25;0.1)	-4.6(25;0.1)	6.75
		Pi-H_3_	1.99(25;0.1)	8.7(25;0.1)	1.99
		Pi-Mg	3.4		3.4
		Pi-HMg	1.8		1.8
		Pi-H2Mg	0.7		0.7
		Pi-K	0.54(25;0.5)	15.0(25.0;0.0)	0.60
		Pi-HK	0.5(25;0.1)		0.5
		Pi-H_2_K	0.3(25;0.0)		0.19
		Pi-HK_2_	11.24(25;0.0)		11.13
		Pi-K_2_	0.83(25;0.0)		0.72
		Pi-Na	1.43(25;0.0)	8.0 (25.0;0.0)	1.32
		Pi-HNa	0.61(25;0.1)		0.61
		Pi-NaH_2_	0.3(25;0.0)		0.19
		Pi-Na_2_	1.16(25;0.0)		1.05
		Pi-Na_2_H	10.73(25;0.0)		10.62
Inosine monophosphate	8.0E-6	IMP-H	6.34(25;0.1)	0.6(25;0.1)	6.34
		IMP-H_2_	1.3(25;0.1)		1.3
		IMP-H_2_	0.4(25;0.1)		0.4
		IMP-Mg	1.68(25;0.1)		1.68
Adenosine monophosphate	8.0E-6	AMP-H	6.29(25;0.1)	0.8(25;0.1)	6.2
		AMP-H_2_	3.8(25;0.1)	-4.0(25;0.1)	3.8
		AMP-Mg	1.97(25;0.1)	1.6(25;0.1)	1.97
		AMP-K	0.7(25;0.1)		0.7
		AMP-Na	0.88(25;0.1)		0.88
Ammonia	0.01	NH_3_-H	9.26(25;0.1)	-12.4(25;0.1)	9.26
		NH_3_-Mg	0.24(25;0.002)		0.15
Adenosine diphosphate	0.0135	ADP-H	6.38(25;0.1)	0.6(25;0.1)	6.38
		ADP-H_2_	3.87(25;0.1)	-4.0(25;0.1)	3.87
		ADP-H_3_	1.8(25;0.0)	2.6(25;0.0)	1.69
		ADP-Mg	3.23(25;0.1)	3.7(25;0.1)	3.23
		ADP-HMg	1.59(25;0.1)	1.8(25;0.1)	1.59
		ADP-Mg_2_	1.7(25;0.0)	3.2(25;0.0)	1.59
		ADP-K	1.00(25;0.1)		1.00
		ADP-Na	1.12(25;0.1)		1.12
Adenosine triphosphate	10.0	ATP-H	6.48(25;0.1)	2.0(25;0.1)	6.48
		ATP-H_2_	3.99(25;0.1)	-3.6(25;0.1)	3.99
		ATP-H_3_	1.9(25;0.1)	1.5(25;0.0)	1.9
		ATP-H_4_			1.0*
		ATP-Mg	4.10(25;0.1)	4.4(25;0.1)	4.10
		ATP-HMg	2.32(25;0.1)	2.3(25;0.1)	2.32
		ATP-Mg_2_	1.7(25;0.1)	5.0(25;0.0)	1.7
		ATP-K	1.17(25;0.1)	0.3(25;0.1)	1.17
		ATP-Na	1.31(25;0.1)	-0.2(25;0.1)	1.31
Creatine phosphate	32.0	CrP-H			14.3*
		CrP-H_2_			4.5*
		CrP-H_3_			2.7*
		CrP-H4			2.0*
		CrP-HMg			1.6*
		CrP-K			0.74*
		CrP-HK			0.31*
		CrP-H_2_K			-0.13*
Creatine	5.4	Cr-H			14.3*
		Cr-H_2_	2.63(25;0.0)	-4.0(25;0.0)	2.52
***Glycogenolysis and Glycolysis***					
Glucose	0.1	Glc-H	12.28(25;0.0)	-36(25;0.0)	12.17
Glucose-1-phosphate	0.11	G1P-H	6.09(25;0.1)	1.5(25;0.1)	6.09
		G1P-H_2_	1.5(25;0.1)		1.5
		G1P-Mg	2.48(25;0.0)	12.0(25;0.0)	2.37
Glucose-6-phosphate	2.43	G6P-H			6.11
Fructose-6-phosphate	0.38	F6P-H	5.89(25;0.1)		5.89
		F6P-H_2_	1.1(25;0.1)		1.1
Fructose-1,6-bisphosphate	0.2	F1,6P-H	6.64(25;0.1)		6.64
		F1,6P-H_2_	5.92(25;0.1)		5.92
		F1,6P-H_3_	2.32(25;0.1)		2.32
		F1,6P-H4	2.06(25;0.1)		2.06
		F1,6P-Mg	2.7(25;0.1)		2.7
		F1,6P-HMg	2.12(25;0.1)		2.12
Dihydroxyacetone phosphate	0.4	DHP-H	5.9(25;0.1)		5.9
		DHP-Mg	1.57(25;0.1)		1.57
Glyceraldehyde-3-phosphate	0.86	G3P-H			6.45*
1,3 bisphosphoglyceric acid	0.4	1,3BPG-H			7.5*
3-phosphoglyceric acid	0.4	3PG-H			6.21*
2-phosphoglyceric acid	0.4	2PG-H	7.0(25;0.1)		7.0*
		2PG-Mg	2.45(25;0.1)		2.45*
		2PG-K	1.18(25;0.1)		1.18*
Phosphoenolpyruvic acid	0.4	PEP-H	6.35 (25;0.1)		6.35
		PEP-H_2_	3.45(25;0.1)		3.45
		PEP-Mg	2.26(25;0.1)		2.26
		PEP-K	1.08(25;0.1)		1.08
Pyruvic acid	0.27	PYR-H	2.26(25;0.1)	-12.8(25;0.1)	2.26
		PYR-Mg	1.1(25;0.1)		1.1
Lactic acid	1.4	La-H	3.67(25;0.1)	0.33(25;0.0)	3.67
		La-Mg	0.98(25;0.1)		0.98

Unless indicated, all data were from the NIST data base

^#^ Total concentrations expressed mmol/L muscle water = (mmol/kg wet wt. / 0.74) = ((mmol/kg dry wt. / 4.1) / 0.74))

* Data obtained from prior published works as explained in text.

Concentrations of metabolites in resting muscle were obtained from original research, as identified in Table 1. As will be explained these concentrations were used in computations of metabolite-cation complex concentrations, which were then converted to fractions and thereby rendering absolute metabolite concentrations redundant for the purposes of this manuscript.

### Influence of temperature, ionic strength and enthalpy of dissociation

The pK is influenced by temperature, ionic strength and the enthalpy of dissociation, as revealed in Eqs 11–13 [1,14]. Although Vinnakota et al. [2] and Kushmerick et al. [1] corrected their pK_M+_ data for temperature, ionic strength and enthalpy of dissociation (ΔH) for their research questions, the corrected pK_M+_ data were not reported. Nevertheless, it is important to note that Alberty [14] has rationalized that the thermodynamic properties of dilute aqueous solutions in biological milieu can be regarded as being independent of temperature. This is logical, as inspection of Eq 11 reveals that the right-hand side of the equation is appreciably small across a range of enthalpy values noted in Table 1. For example, the metabolite-cation complex of ATPH^-3^ has a pK = 6.48 (T = 25°C, IS = 0.1) and ΔH = 2.0 kJ/M (T = 25°C, IS = 0.1). How does this pK change for a muscle temperature of 40°C, as is typical during intense exercise? Use of Eq 1 reveals that the ATP-H pK would increase by 0.001679. Given that reference values for pK data are reported with precision to two decimal places, there is no rationale for temperature correction of these thermodynamic constants to match *in-vivo* conditions for contracting skeletal muscle.

As not all reference data from the NIST database was presented for ionic strength conditions of 0.1 M, minor correction had to occur for the pH data of these metabolite-cation complexes to make all reference values consistent to standard conditions (T = 25°C, IS = 0.1). For these metabolite-cation species, the pK _M+_ was corrected for ionic strength of 0.1 M using Eq 13 as explained by Alberty [14] and Vinakota et al. [2].

$$pKa_{T2}=pKa_{T1}+\left(\frac{1}{T2}-\frac{1}{T1}\right)\frac{\mathrm{\Delta H}Η{}^{\circ}}{2.303\;R}$$ (11)

$$\mathrm{\Delta H}Η{}^{\circ}=\mathrm{\Delta H}Η{}^{\circ}\left(I=0\right)+\frac{1.4775I^{1/2}\sum v_{j}z_{j}{}^{2}}{1+1.6I^{1/2}}$$ (12)

Where *v*_*j*_ = stoichiometric coefficient for cation j; *z*_*j*_ = charge on cation j.

$$pKa\left(I\right)=pKa\left(I_{1}\right)+\frac{1.17582}{2.303}\left(\frac{I_{1}{}^{1/2}}{1+1.6I_{1}{}^{1/2}}-\frac{I^{1/2}}{1+1.6I^{1/2}}\right)$$ (13)

Where *I*_1_ = reference ionic strength (0.1 M)

Additional concern over correction of ΔH is that only 40% ((30/75)*100) of the metabolite-cation complexes involved in this investigation have data in the NIST database for ΔH. Without this data, there can be no correction of pK for temperature (Eq 11) or ΔH for ionic strength (Eq 12). With correction, there would be some metabolites of reactions with adjusted data, and others (most) without. Finally, and as clearly revealed within the NIST database [13], the dissociation constants are influenced not just by the ionic strength, but also the ionic composition of the reaction milieu at a given ionic strength. No experimental ionic strength condition is truly representative of the intracellular biological milieu, and it is also clear that during times of metabolic stress, each of ionic strength and cation concentrations change. Consequently, there is doubt as to the validity, or improved accuracy, of any adjustment. The most prudent approach is to select the most representative ionic strength condition for a given metabolite ionic association complex from the NIST data base, and report these conditions.

### Computations of competitive cation binding

Based on the data of Table 1, the concentrations of each ligand (L) to cation (A^a+^) complex was computed using an α-equation common to analytical chemistry, as derived in Eqs 14–24. As will be explained, the free H^+^ concentration was treated as a variable in computations across the physiological range of muscle pH (6.0 to 7.0), and the following constants were assumed for Mg^2+^ (0.6 mM), K^+^ (120 mM) and Na^+^ (15 mM) based on prior research and the predominant conditions from research within the NIST database [1,2,12].

The total concentration of a ligand (L_tot_) in solution with a mix of H^+^ and metal cations (A^m+^, B ^m+^, …) can be written as,

$$L_{tot}=\left[L^{n-}\right]+\left[HL^{1-n}\right]+\ldots +\left[H_{n}L\right]+\left[AL^{a-n}\right]+\left[AH_{2}L^{2+a-n}\right]+\left[BHL^{1+b-n}\right]+\ldots .$$ (14)

A dissociation constant was written for each ligand to cation(s) species.

$$K_{H}=\frac{\left[HL^{1-n}\right]}{\left[H^{+}\right]\left[L^{n-}\right]}$$ (15)

$$K_{H2}=\frac{\left[H_{2}L^{2-n}\right]}{{\left[H^{+}\right]}^{2}\left[L^{n-}\right]}$$ (16)

$$K_{A}=\frac{\left[AL^{a-n}\right]}{\left[A^{a+}\right]\left[L^{n-}\right]}$$ (17)

$$K_{AH}=\frac{\left[AHL^{1+a-n}\right]}{\left[A^{a+}\right]\left[H^{+}\right]\left[L^{n-}\right]}$$ (18)

$$K_{AHH}=\frac{\left[AH_{2}L^{1+a-n}\right]}{\left[A^{a+}\right]{\left[H^{+}\right]}^{2}\left[L^{n-}\right]}$$ (19)

$$K_{AAH}=\frac{\left[A_{2}HL^{1+a-n}\right]}{{\left[A^{a+}\right]}^{2}\left[H^{+}\right]\left[L^{n-}\right]}$$ (20)

Each dissociation constant was then solved for the cation-ligand species.

$$\left[HL^{1-n}\right]=K_{H}\left[H^{+}\right]\left[L^{n-}\right]$$ (21)

The solution for each cation-ligand species of Eq 21 was then substituted into Eq 14.

$$L_{tot}=\left[L^{n-}\right]+K_{H}\left[H^{+}\right]\left[L^{n-}\right]+K_{Hn}{\left[H^{+}\right]}^{n}\left[L^{n-}\right]+K_{A}\left[A^{a+}\right]\left[L^{n-}\right]+K_{AH}\left[A^{a+}\right]\left[H^{+}\right]\left[L^{n-}\right]+\ldots .$$ (22)

When each side of Eq 22 is divided by the free ligand concentration ([*L*^*n*−^]) (Eq 23), and the reciprocal of each side was then taken, an “α equation” was derived (Eq 24).

$$\frac{L_{tot}}{\left[L^{n-}\right]}=1+K_{H}\left[H^{+}\right]+\ldots .+K_{Hn}{\left[H^{+}\right]}^{n}+K_{A}\left[A^{a+}\right]+K_{AH}\left[A^{a+}\right]\left[H^{+}\right]+\ldots .$$ (23)

$$\alpha_{-n}=\frac{\left[L^{n-}\right]}{L_{tot}}=\frac{1}{1+K_{H}\left[H^{+}\right]+\ldots .+K_{Hn}{\left[H^{+}\right]}^{n}+K_{A}\left[A^{a+}\right]+K_{AH}\left[A^{a+}\right]\left[H^{+}\right]+..}$$ (24)

(Note that for multiple cation binding, *K*_*AH*_ in Eq 20 refers to *K*_H_*K_*AH*_, etc)

The *α*_−*n*_ is the fraction of the total ligand present in the cation-free form. Once this fraction was computed, then [*L*^*n*−^] was calculated using Eq 25.

$$\left[L^{n-}\right]=\alpha_{-n}L_{tot}$$ (25)

Now that [*L*^*n*−^] was known, Eq 25 was expressed for each cation complexed and protonated species so that the only unknown was the cation complexed ligand concentration (Eq 26).

$$K_{A}\left[A^{a+}\right]\left[L^{n-}\right]=\left[AL^{{}_{a-n}}\right]$$ (26)

The use of this “α equation” provided the mathematical derivation presumed to be operational in the MathLab application of Kushmerick [1] and Vinnakota et al. [2]. The metabolite-cation complex fractions for cellular pH ranging from 6.0 to 7.0 in 0.1 increments were then computed for the metabolites and accompanied data of Table 1 (including cation “free” metabolite complexes) (Eq 27) using a commercial spreadsheet program (Excel, Microsoft Corporation, Seattle, WA). For comparison, data was also computed for the main metabolite-H^+^ complex fraction without correction for multiple cation binding. The H^+^ fractional content of the metabolite-cation complexes was then computed by summing all H^+^ fractions, adjusted by H^+^ content of each metabolite-cation complex, to derive the H^+^ fraction of each metabolite (Eq 28). The data were imported into a graphics program (Prism, GraphPad Software, San Diego, CA) for nonlinear curve-fitting to derive equations (2^nd^, 3^rd^ or 4^th^ order polynomial) for the fraction of each metabolite-cation complex for the previously stated pH range.

$$A_{n}H_{n}L^{-}{}_{f}=\frac{\left[A_{n}H_{n}L^{-}\right]}{\left[L^{-}{}_{tot}\right]}$$ (27)

$$H_{f}=A_{n}HL^{-}{}_{f}+2\left(A_{n}H_{2}L^{-}{}_{f}\right)+3\left(A_{n}H_{3}L^{-}{}_{f}\right)+4\left(A_{n}H_{4}L^{-}\right)$$ (28)

### Proton coefficients for specific reactions

The reactions of Table 2 were used to compute the H^+^ balance (coefficient) for each reaction, based on the fraction of cation-metabolite species containing H^+^ computed from Eqs 18–24. Computations for H^+^ coefficients are not as simple as depicted by Kushmerick et al. [1] or Vinnakota et al. [2], as the change in H^+^ coefficient can be from differences in ionization between substrates and products, consumption from H^+^ in solution, or production from covalent modification of substrates (e.g. hydrolysis reactions). Each condition requires a unique formula. The formula used for each reaction is presented in Table 3.

**Table 2:** The chemical reactions of muscle phosphagen and glycolytic energy systems*.

Reaction	Enzyme	ΔH^+^
***Phosphagen System***		
HCrP + ADP + H^+^ ↔ Cr + ATP	Creatine kinase	+1
ADP + ADP ↔ ATP + AMP	Adenylate kinase	0
AMP + H^+^ ↔ IMP + NH_4_	AMP deaminase	+1
ATP + H_2_O ↔ ADP + Pi +H^+^	ATPase	-1
***Glycogenolysis***		
Glycogen(n) + HP ↔ Glycogen(n-1) + G1P	Phosphorylase	0
G1P ↔ G6P	Phosphogluco mutase	0
***Glycolysis***		
Glucose + ATP ↔ G6P + ADP + H^+^	Hexokinase	-1
G6P ↔ F6P	Glucose-6-phosphate isomerase	0
F6P + ATP ↔ F1,6P + ADP + H^+^	Phosphofructokinase	-1
F1,6P ↔ DHP + G3P	Aldolase	0
DHP ↔ G3P	Triosephosphate Isomerase	0
G3P + HPi + NAD+ ↔ 1,3BPG + NADH + H^+^	Glyceraldehyde-3-phosphate dehydrogenase	-1
1,3BPG + ADP ↔ 3PG + ATP	Phosphoglycerate kinase	0
3PG ↔ 2PG	Phosphoglycerate mutase	0
2PG + ADP ↔ PEP	Enolase	0
PEP + ADP + H+ ↔ Pyr + ATP	Pyruvate kinase	+1
***Lactate Production***		
Pyr + NADH + H^+^ ↔ La + NAD^+^	Lactate dehydrogenase	+1

* ΔH^+^ = reference proton load value prior to adjustment for partial ionization (-’ve = proton release, +‘ve = proton consumption); See Table 1 for metabolite abbreviation definitions.

**Table 3:** Proton coefficients for specific reactions of non-mitochondrial energy metabolism.

Reaction
***Phosphagen System***
$\Delta_{r}{CK}_{H^{+}}=\left(\sum {ATP}_{H^{+}}^{}+\sum {Cr}_{H^{+}}^{}\right)-\left(\sum {CrP}_{H^{+}}^{}+\sum {ADP}_{H^{+}}^{}\right)$
$\Delta_{r}{AK}_{H^{+}}=\left(\sum {ATP}_{H^{+}}^{}+\sum {AMP}_{H^{+}}^{}\right)-\left(\sum {ADP}_{H^{+}}^{}+\sum {ADP}_{H^{+}}^{}\right)$
$\Delta_{r}{AMPD}_{H^{+}}=\left(\sum {NH_{3}H}_{H^{+}}^{}+\sum {IMP}_{H^{+}}^{}\right)-\sum {AMP}_{H^{+}}^{}$
$\Delta_{r}{ATPase}_{H^{+}}=\left(\sum {ADP}_{H^{+}}^{}+\sum {Pi}_{H^{+}}^{}\right)-\left(\sum {ATP}_{H^{+}}^{}+2\right)$
***Glycogenolysis***
$\Delta_{r}{Phosph}_{H^{+}}=\left(\sum {G1P}_{H^{+}}^{}-\sum {Pi}_{H^{+}}^{}\right)+1$
$\Delta_{r}{PGM}_{H^{+}}=\sum {G6P}_{H^{+}}^{}-\sum {G1P}_{H^{+}}^{}$
***Glycolysis***
$\Delta_{r}{HK}_{H^{+}}=\left(\sum {G6P}_{H^{+}}^{}+\sum {ADP}_{H^{+}}^{}\right)-\left(\sum {Glu}_{H^{+}}^{}+\sum {ATP}_{H^{+}}^{}\right)$
$\Delta_{r}{PGI}_{H^{+}}=\sum {F6P}_{H^{+}}^{}-\sum {G6P}_{H^{+}}^{}$
$\Delta_{r}{PFK}_{H^{+}}=\left(\sum {F1,6P}_{H^{+}}^{}+\sum {ADP}_{H^{+}}^{}\right)-\left(\sum {F6P}_{H^{+}}^{}+\sum {ATP}_{H^{+}}^{}\right)-1$
$\Delta_{r}{Ald}_{H}=\left(\sum {DHP}_{H^{+}}^{}+\sum {G3P}_{H^{+}}^{}\right)-\sum {F1,6P}_{H^{+}}^{}$
$\Delta_{r}{TPI}_{H^{+}}=\sum {G3P}_{H^{+}}^{}-\sum {DHP}_{H^{+}}^{}$
$\Delta_{r}{G3PDH}_{H^{+}}=\sum {1,3BPG}_{H^{+}}^{}-\left(\sum {GsP}_{H^{+}}^{}+\sum {Pi}_{H^{+}}^{}\right)$
$\Delta_{r}{PGK}_{H^{+}}=\left(\sum {3PG}_{H^{+}}^{}+\sum {ATP}_{H^{+}}^{}\right)-\left(\sum {1,3BPG}_{H^{+}}^{}+\sum {ADP}_{H^{+}}^{}\right)$
$\Delta_{r}{PGM}_{H^{+}}=\sum {2PG}_{H^{+}}^{}-\sum {3PG}_{H^{+}}^{}$
$\Delta_{r}{Enol}_{H^{+}}=\sum {PEP}_{H^{+}}^{}-\sum {2PG}_{H^{+}}^{}$
$\Delta_{r}{PK}_{H^{+}}=\left(\sum {Pyr}_{H^{+}}^{}+\sum {ATP}_{H^{+}}^{}\right)-\left(\sum {PEP}_{H^{+}}^{}+\sum {ADP}_{H^{+}}^{}\right)+1$
***Lactate Production***
$\Delta_{r}{LDH}_{H^{+}}=\left(\sum {La}_{H^{+}}^{}-\sum {Pyr}_{H^{+}}^{}\right)+1$

Computations of the H^+^ coefficients for chemical reactions provided negative coefficients for H^+^ release and positive coefficients for H^+^ consumption (metabolic buffering), consistent with thermodynamic computations. Data of the pH profile of the H^+^ coefficient of each reaction of Table 2 were imported into a graphics curve-fitting program (Prism, GraphPad Software, San Diego, CA). Nonlinear curve fitting was applied to each data set to derive equations (2^nd^, 3^rd^ or 4^th^ order polynomial) for the proton coefficient of each reaction for the physiological pH (x-variable) range from 6.0 to 7.0.

### Proton coefficient for glycolysis

Computations were made for the H^+^ coefficient of glycolysis when starting with glycogen vs. glucose by summing the net proton balance of each reaction of the pathway. For the reactions of the 3-carbon glycolytic intermediates, the reaction proton coefficient was doubled to balance carbon flux between phases 1 and 2 of glycolysis. Added calculations were also made for glycolysis ending in lactate production, as well as for phase 1 (glycogen or glucose to DHP + G3P) and 2 of glycolysis (DHP or G3P to pyruvate or lactate). Each data set was fit with nonlinear curve fitting to derive equations (2^nd^, 3^rd^ or 4^th^ order polynomial) for the net H^+^ coefficient for the series of reactions the physiological pH (x-variable) range from 6.0 to 7.0.

## Results and discussion

Data presented from this research can be used as reference data for the pH dependent fractional H^+^ binding to metabolites and the pH dependent H^+^ coefficients of all reactions of non-mitochondrial energy metabolism. Consequently, as much data as feasible is reported for each metabolite and reaction. In addition, supporting information is presented in three different data files; S1 File for an Excel spreadsheet of all data used to calculate H^+^ exchange across the pH range of 6.0 to 7.0; S2 File for a Prism file of data and curve fitting equations for the H^+^ exchange of metabolites and chemical reactions of the phosphagen and glycolytic energy systems; and S3 File for data and curve fitting equations for the H^+^ exchange of all metabolites.

### Preliminary data

In order to compute fractional cation binding and show differences to singular H^+^ based dissociation, reference data was acquired and computed as presented in Tables 1 and 2. Table 1 presents data for intramuscular concentrations of intermediates, and the association constants and enthalpy of dissociation for select metabolite and cation complexes for the three intramuscular catabolic energy systems. Table 2 presents the summary reactions of the phosphagen and glycolytic energy systems (non-mitochondrial energy catabolism) with their enzymes and generic referenced H^+^ exchange coefficients.

### Metabolite-Cation binding

**Fig 1:**
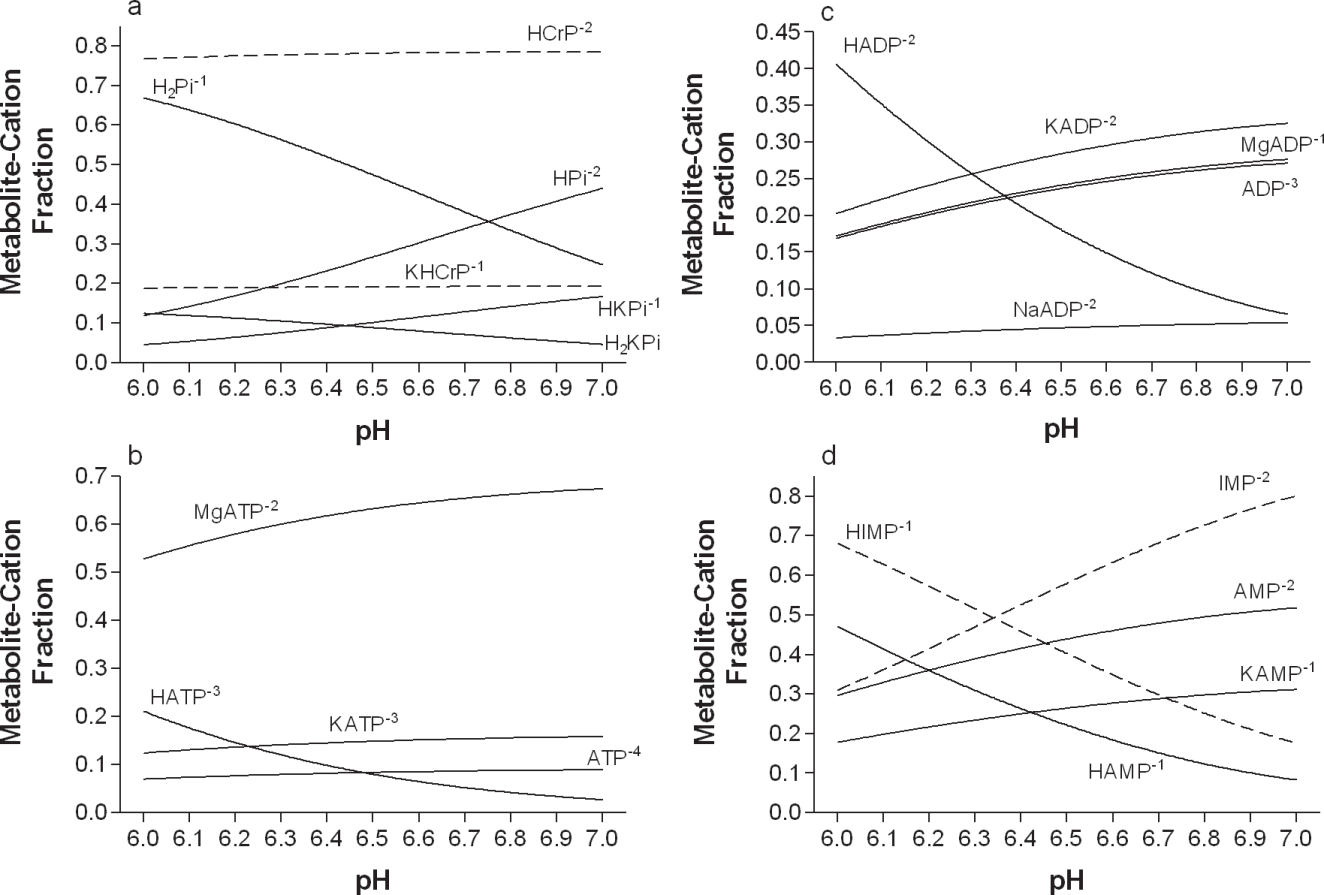
**Metabolite-cation complex fractions for the main phosphorylated metabolites of intermediary metabolism, grouped by a) the creatine kinase reaction, b) ATP, c) ADP, and d) IMP and AMP.** Polynomial equations are presented in Table 4.

Table 3 presents the formulae used to compute the net H+ exchange for the reactions of Table 2. The pH dependence of the fraction of metabolite-cation complexes of the metabolites of Table 1 are presented in Table 4. Fractions of metabolite-cation complexes at pH 6.0 and 7.0 are presented for computations based on competitive binding between H^+^ and Mg^2+^, K^+^ and Na^+^. For comparison, data for the main metabolite-H^+^ complex are also provided based on non-competitive binding computation. Differences between competitive and non-competitive binding computations reveal the error of using metabolite specific pK_M+_ data in computations. Exponential equations are provided for calculations of the fraction of bound metabolite-cation complexes across the pH range of 6.0 to 7.0. For visual clarity, the main metabolite-cation complexes of interest, based on large or highly pH dependent H^+^ binding, are presented in Fig 1A–1D.

**Table 4:** The binding fraction and sum of H^+^ binding (∑H^+^) of metabolite-cation complexes for non-mitochondrial muscle energy metabolism.

	Non-competitive^#^			Multiple Competitive Cation Binding		
Complex	pH = 6.0	pH = 7.0		pH = 6.0	pH = 7.0	Polynomial*
*Phosphagen*						
Pi^-3^				3.0581E-7	1.1323E-5	A = -0.002923; B = 0.001429; C = -0.0002332; D = 1.271E-5
Pi-H				0.1190	0.4405	A = 34.78; B = -16.31; C = 2.513; D = -0.1263
Pi-H_2_	0.8490	0.3599		0.6690	0.2477	A = -44.77; B = 21.37; C = -3.293; D = 0.1655
Pi-H_3_				6.5382E-5	2.4208E-6	A = 0.02009; B = -0.008669; C = 0.00125; D = -6.014E-5
Pi-Mg				4.6090E-7	1.7065E-5	A = -0.004405; B = 0.002154; C = -0.0003515; D = 1.915E-5
Pi-HMg				4.5041E-3	1.6676E-2	A = 1.317; B = -0.6174; C = 0.09513; D = -0.00478
Pi-H_2_Mg				2.0119E-3	7.4491E-4	A = -0.1346; B = 0.06428; C = -0.009903; D = 0.0004976
Pi-K				1.4610E-7	5.4092E-6	A = -0.001396; B = 0.0006827; C = -0.0001114; D = 6.071E-6
Pi-HK				4.5148E-2	0.1672	A = 13.2; B = -6.189; C = 0.9535; D = -0.04792
Pi-H_2_K				0.1243	4.6040E-2	A = -8.32; B = 3.973; C = -0.6121; D = 0.03076
Pi-HK_2_				1.2411E-2	4.5953E-2	A = 3.629; B = -1.701; C = 0.2621; D = -0.01317
Pi-K_2_				9.2001E-8	3.4065E-6	A = -0.0008794; B = 0.0004299; C = -7.017E-5; D = 3.823E-6
Pi-Na				9.5840E-8	3.5485E-6	A = -0.000916; B = 0.0004479; C = -7.309E-5; D = 3.982E-6
Pi-HNa				7.2702E-3	2.6918E-2	A = 2.126; B = -0.9966; C = 0.1535; D = -0.007716
Pi-NaH_2_				1.5543E-2	5.7550E-3	A = -1.04; B = 0.4966; C = -0.07651; D = 0.003845
Pi-Na_2_				1.6130E-8	5.9722E-7	A = -0.0001542; B = 7.538E-5; C = -1.23E-5; D = 6.702E-7
Pi-Na_2_H				6.7242E-4	2.4896E-3	A = 0.1966; B = -0.09217; C = 0.0142; D = -0.0007136
***∑H (Fraction)***				***1*.*8111***	***1*.*3002***	*A = -53*.*21; B = 25*.*89; C = -3*.*988; D = 0*.*2004*
IMP^-1^				0.3109	0.8016	A = 43.5; B = -21.53; C = 3.495; D = -0.1843
IMP-H	0.6863		0.1795	0.6802	0.1754	A = -43.75; B = 22.15; C = -3.595; D = 0.1896
IMP-H_2_				1.3571E-5	3.4991E-7	A = 0.00513; B = -0.002233; C = 0.0003244; D = -1.573E-5
IMP-H_3_				3.4089E-11	8.7894E-14	A = 2.853E-8; B = -1.276E-8; C = 1.901E-9; D = -9.441E-11
IMP-Mg				3.4089E-11	8.7894E-14	A = 1.249; B = -0.6183; C = 0.1004; D = -0.005293
***∑H (Fraction)***				***0*.*6802***	***0*.*1754***	*A = -43*.*74; B = 22*.*15; C = -3*.*595; D = 0*.*1896*
AMP^-1^				0.2971	0.5182	A = -2.627; B = 0.1279; C = 0.1438; D = -0.01398
AMP-H	0.6131		0.1368	0.4708	0.0821	A = 4.207; B = 0.4092; C = -0.3479; D = 0.02933
AMP-H_2_				0.4708	0.0821	A = 1.446; B = -0.6357; C = 0.09325; D = -0.004562
AMP-Mg				2.9708E-2	2.9016E-2	A = -0.1471; B = 0.007162; C = 0.008052; D = -0.0007831
AMP-K				0.1787	0.3116	A = -1.58; B = 0.07694; C = 0.08648; D = -0.008411
AMP-Na				3.3804E-2	5.8963E-2	A = -0.2989; B = 0.01456; C = 0.01636; D = -0.001591
***∑H (Fraction)***				***0*.*4768***	***0*.*0822***	*A = 7*.*098; B = -0*.*8623; C = -0*.*1614; D = 0*.*02021*
NH_3_^-1^				5.4924E-4	5.4653E-4	A = -0.8459; B = 0.4163; C = -0.06853; D = 0.003775
NH_3_-H	0.9995		0.9945	0.9994	0.9945	A = 1.847; B = -0.4167; C = 0.06859; D = -0.003779
NH_3_-Mg				4.6549E-7	4.6320E-6	A = -0.0007169; B = 0.0003529; C = -5.808E-5; D = 3.2E-6
***∑****H (Fraction)*				*0*.*9994*	*0*.*9945*	*A = 1*.*847; B = -0*.*4167; C = 0*.*06859; D = -0*.*003779*
ADP^-4^				0.1691	0.2713	A = -4.365; B = 1.483; C = -0.1452; D = 0.003993
ADP-H	0.7058		0.1935	0.4057	6.5077E-2	A = 14.14; B = -4.321; C = 0.3905; D = -0.008627
ADP-H_2_				3.0074E-3	4.8242E-5	A = 1.546; B = -0.6814; C = 0.1001; D = -0.004908
ADP-H_3_				1.4729E-7	2.3628E-10	A = 0.0001375; B = -6.164E-5; C = 9.211E-6; D = -4.586E-7
ADP-Mg				0.1723	0.2764	A = -4.447; B = 1.511; C = -0.148; D = 0.004068
ADP-HMg				9.4670E-3	1.5191E-3	A = 0.3301; B = -0.1009; C = 0.009114; D = -0.0002014
ADP-Mg_2_				4.0224E-3	6.453E-3	A = -0.1038; B = 0.03528; C = -0.003455; D = 9.497E-5
ADP-K				0.2029	0.3255	A = -5.237; B = 1.78; C = -0.1743; D = 0.004792
ADP-Na				3.3441E-2	5.3644E-2	A = -0.863; B = 0.2933; C = -0.02872; D = 0.0007896
***∑H (Fraction)***				***0*.*4212***	***6*.*6669E-2***	*A = 17*.*56; B = -5*.*785; C = 0*.*5999; D = -0*.*01865*
ATP^-4^				6.9979E-2	8.9269E-2	A = -2.695; B = 1.14; C = -0.1562; D = 0.007161
ATP-H	0.7512		0.2319	0.2113	2.6959E-2	A = 25.81; B = -10.52; C = 1.436; D = -0.0656
ATP-H_2_				2.0652E-3	2.6345E-5	A = 1.231; B = -0.545; C = 0.0805; D = -0.003964
ATP-H_3_				1.6405E-7	2.0927E-10	A = 0.0001619; B = -7.273E-5; C = 1.088E-5; D = -5.427E-7
ATP-H_4_				1.6405E-12	2.0927E-16	A = 2.034E-9; B = -9.184E-10; C = 1.382E-10; D = -6.924E-12
ATP-Mg				0.5286	0.6743	A = -20.36; B = 8.611; C = -1.18; D = 0.05409
ATP-HMg				2.6492E-2	3.3379E-3	A = 3.235; B = -1.319; C = 0.18; D = -0.008223
ATP-Mg_2_				1.5895E-2	2.0203E-2	A = -0.6122; B = 0.259; C = -0.03547; D = 0.001627
ATP-K				0.12442	0.1584	A = -4.783; B = 2.024; C = -0.2772; D = 0.01271
ATP-Na				2.1432E-2	2.7340E-2	A = -0.8254; B = 0.3492; C = -0.04783; D = 0.002193
***∑H (Fraction)***				***0*.*2420***	***0*.*0304***	*A = 31*.*5; B = -12*.*93; C = 1*.*777; D = -0*.*08175*
CrP^-3^				3.8454E-9	3.9391E-8	A = -6.198E-6; B = 3.049E-6; C = -5.016E-7; D = 2.762E-8
CrP-H	0.9693		0.9968	0.7673	0.7860	A = -3.893; B = 2.003; C = -0.2867; D = 0.01372
CrP-H_2_				2.4263E-2	2.4854E-3	A = 5.447; B = -2.33; C = 0.3335; D = -0.01596
CrP-H_3_				1.2160E-5	1.2457E-7	A = 0.008278; B = -0.003682; C = 0.0005461; D = -2.7E-5
CrP-H_4_				1.2160E-9	1.2465E-12	A = 1.267E-6; B = -5.698E-7; C = 8.539E-8; D = -4.263E-9
CrP-HMg				1.8327E-2	1.8774E-2	A = -0.09299; B = 0.04784; C = -0.006848; D = 0.0003278
CrP-K				2.5358E-9	2.5976E-8	A = -4.088E-6; B = 2.011E-6; C = -3.308E-7; D = 1.821E-8
CrP-HK				0.1880	0.1926	A = -0.9539; B = 0.4907; C = -0.07024; D = 0.003362
CrP-H_2_K				2.158E-3	2.2211E-4	A = 0.4846; B = -0.2073; C = 0.02967; D = -0.00142
***∑H (Fraction)***				***1*.*0264***	***1*.*0027***	*A = 6*.*948; B = -2*.*545; C = 0*.*3643; D = -0*.*01744*
Cr^-1^				5.0119E-5	5.0119E-5	A = 2.733E-5; B = -1.786E-5; C = -4.387E-6; D = -4.807–7; E = 1.984E-8
Cr-H	0.9999		0.9999	0.9999	0.9999	A = 1.002; B = -1.142E-3; C = -2.642E-4; D = -2.712–5; E = 1.042E-6
Cr-H2				1.1949E-16	1.1949E-17	A = 1.122E-13; B = -6.392E-14; C = -1.372E-14; D = -1.313E-15; E = 4.729E-17
***∑H (Fraction)***				***1*.*008***	***1*.*008***	*A = 1*.*004; B = 2*.*197E-3; C = -5*.*145E-4; D = 5*.*349–5; E = -2*.*084E-6*
***Glycolysis***						
Glc^-1^				6.7608E-7	6.7608E-6	A = -0.00061; B = 0.000522; C = -8.587E-5; D = 4.728E-6
Glc-H	0.9999		0.9999	0.9999	0.9999	A = 1.001; B = -0.0005051; C = 8.317E-5; D = -4.585E-6
***∑H (Fraction)***				***0*.*9999***	***0*.*9999***	*A = 1*.*001; B = -0*.*0005051; C = 8*.*317E-5; D = -4*.*585E-6*
G1P^-1^				0.4218	0.7913	A = 5.187; B = -4.085; C = 0.876; D = -0.05459
G1P-H	0.5516		0.1095	0.5189	0.0973	A = -4.924; B = 4.663; C = -0.9997; D = 0.0623
G1P-H_2_				1.6410E-5	3.0790E-7	A = 0.007625; B = -0.003347; C = 0.0004901; D = -2.394E-5
G1P-Mg				5.9323E-2	0.1113	A = 0.7296; B = -0.5746; C = 0.1232; D = -0.007679
***∑H (Fraction)***				***0*.*5189***	***0*.*0973***	*A = -4*.*917; B = 4*.*66; C = -0*.*9992; D = 0*.*06227*
G6P^-1^				0.4370	0.8859	A = 16.52; B = -9.52; C = 1.734; D = -0.09897
G6P-H	0.5630		0.1141	0.5630	0.1141	A = -15.52; B = 9.52; C = -1.734; D = 0.09897
***∑H (Fraction)***				***0*.*5630***	***0*.*1141***	*A = -15*.*52; B = 9*.*52; C = -1*.*734; D = 0*.*09897*
F6P-1				0.5630	0.9280	A = -10.98; B = 3.216; C = -0.2088; D = -0.00107
F6P-H	0.4370		7.2033E-2	0.4370	7.2033E-2	A = 11.98; B = -3.215; C = 0.2087; D = 0.001079
F6P-H_2_				5.5016E-6	9.0684E-8	A = 0.002784; B = -0.001226; C = 0.0001801; D = -8.821E-6
***∑H (Fraction)***				***0*.*4370***	***0*.*0720***	*A = 11*.*99; B = -3*.*217; C = 0*.*209; D = 0*.*001061*
F1,6P^-4^				0.10371	0.5531	A = 66.06; B = -31.07; C = 4.814; D = -0.2445
F1,6P-H	0.8136		0.3039	0.4527	0.2414	A = -104.6; B = 46.72; C = -6.87; D = 0.3337
F1,6P-H_2_				0.3765	2.0081E-2	A = 27.92; B = -9.98; C = 1.15; D = -0.0419
F1,6P-H_3_				7.8669E-5	4.1955E-7	A = 0.04835; B = -0.02141; C = 0.003159; D = -0.0001555
F1,6P-H_4_				9.0324E-9	4.8171E-12	A = 9.113E-6; B = -4.094E-6; C = 6.127E-7; D = 3.056E-8
F1,6P-Mg				3.1186E-2	0.1663	A = 19.87; B = -9.343; C = 1.447; D = -0.07353
F1,6P-HMg				3.581E-2	1.9096E-2	A = -8.275; B = 3.695; C = -0.5434; D = 0.0264
***∑H (Fraction)***				***1*.*2418***	***0*.*3007***	*A = -56*.*91; B = 30*.*39; C = -5*.*105; D = 0*.*2759*
DHP^-1^				0.5505	0.9077	A = -10.7; B = 3.126; C = -0.2012; D = -0.001206
DHP-H	0.4427		7.3588E-2	0.4372	7.2099E-2	A = 11.94; B = -3.195; C = 0.2057; D = 0.001233
DHP-Mg				1.2271E-2	2.0234E-2	A = -0.2386; B = 0.06968; C = -0.004484; D = 2.688E-5
***∑H (Fraction)***				***0*.*4373***	***7*.*210E-2***	*A = 11*.*94; B = -3*.*195; C = 0*.*2057; D = 0*.*001233*
G3P^-1^				0.2619	0.7801	A = 54.45; B = -26.23; C = 4.155; D = -0.2146
G3P-H	0.7381		0.2199	0.7381	0.2199	A = -53.45; B = 26.23; C = -4.155; D = 0.2146
***∑H (Fraction)***				***0*.*7381***	***0*.*2199***	*A = -53*.*45*.*; B = 26*.*23; C = -4*.*155; D = 0*.*2146*
1,3BPG^-1^				3.0653E-2	0.2403	A = -14.27; B = 7.403; C = -1.288; D = 0.07518
1,3BPG-H	0.9696		0.7597	0.9693	0.7597	A = 15.27; B = -7.403; C = 1.288; D = -0.07518
***∑****H (Fraction)*				***0*.*9693***	***0*.*7597***	*A = 15*.*27; B = -7*.*403*.*; C = 1*.*288; D = -0*.*07518*
3PG^-1^				0.3814	0.8605	A = 30.42; B = -15.81; C = 2.671; D = -0.1452
3PG-H	0.6186		0.1395	0.6186	0.1395	A = -29.42; B = 15.81; C = -2.671; D = 0.1452
***∑H (Fraction)***				***0*.*6186***	***0*.*1395***	*A = -29*.*42; B = 15*.*81; C = -2*.*671; D = 0*.*1452*
2PG^-1^				7.7010E-2	0.2509	A = 19.05; B = -9.065; C = 1.419; D = -0.0725
2PG-H	0.9091		0.5573	0.7701	0.2509	A = -55.86; B = 27.06; C = -4.236; D = 0.2164
2PG-Mg				1.3023E-2	4.2431E-2	A = 3.221; B = -1.533; C = 0.24; D = -0.01226
2PG-K				0.1399	0.4557	A = 34.59; B = -16.46; C = 2.577; D = -0.1317
***∑H (Fraction)***				***0*.*7701***	***0*.*2509***	*A = -55*.*86; B = 27*.*06; C = -4*.*236; D = 0*.*2164*
PEP^-1^				0.2085	0.3603	A = -2.13; B = 0.2359; C = 0.07674; D = -0.008518
PEP-H	0.6912		0.1829	0.4667	8.0650E-2	A = 5.792; B = -0.3185; C = -0.2374; D = 0.02377
PEP-H_2_				1.3153E-3	2.2730E-5	A = 0.6447; B = -0.2836; C = 0.0416; D = -0.002036
PEP-Mg				2.2761E-2	3.3933E-2	A = -0.2326; B = 0.02576; C = 0.008379; D = -0.00093
PEP-K				0.3008	0.5197	A = -3.073; B = 0.3404; C = 0.1107; D = -0.01229
***∑H (Fraction)***				***0*.*4693***	***8*.*070E-2***	*A = 7*.*081*.*; B = -0*.*8856; C = -0*.*1542; D = 0*.*0197*
PYR^-1^				0.9923	0.9925	A = 0.9502; B = 0.01817; C = -0.002608; D = 0.0001251
PYR-H	1.8194E-4		1.8197E-5	1.8057E-4	1.8060E-5	A = 0.0427; B = -0.01832; C = 0.00263; D = -0.0001262
PYR-Mg				7.4956E-3	7.4968E-3	A = 0.007175; B = 0.0001384; C = -1.988E-5; D = 9.545E-7
***∑H (Fraction)***				***1*.*8057E-4***	***1*.*8060E-5***	*A = 0*.*0427; B = -0*.*01832; C = 0*.*00263; D = -0*.*0001262*
La^-1^				0.9897	0.9938	A = -0.08489; B = 0.4629; C = -0.06641; D = 0.003186
La-H	4.6556E-3		4.6752E-4	4.6292E-3	4.6485E-4	A = 1.085; B = -0.4655; C = 0.06679; D = -0.003204
La-Mg				5.6709E-3	5.6947E-3	A = -0.0004883; B = 0.002653; C = -0.0003806; D = 1.826E-5
***∑H (Fraction)***				***4*.*6292E-3***	***4*.*6485E-4***	*A = 1*.*085; B = -0*.*4655; C = 0*.*06679; D = -0*.*003204*

^#^for most prevalent metabolite-H^+^ complex

*Binding fraction = A + Bx + Cx^2^ + Dx^3^ + Ex^4^, x = pH

### Proton coefficients for specific reactions and glycolysis

**Fig 2:**
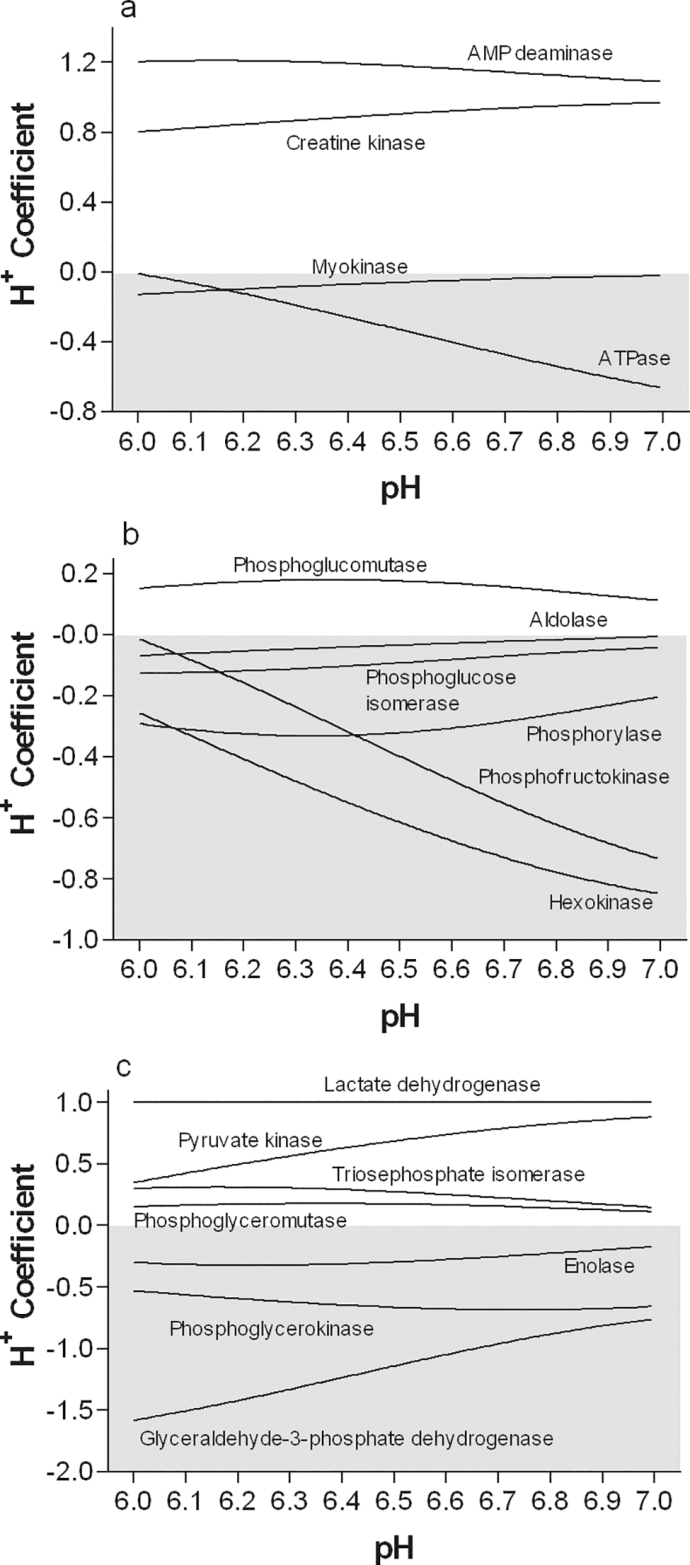
**H**^**+**^ **coefficients for the reactions of a) the phosphagen system, b) phase 1 of glycolysis, and c) phase 2 of glycolysis.–‘ve coefficients (shaded area) = H**^**+**^ **release.** Polynomial equations are presented in Table 5.

**Fig 3:**
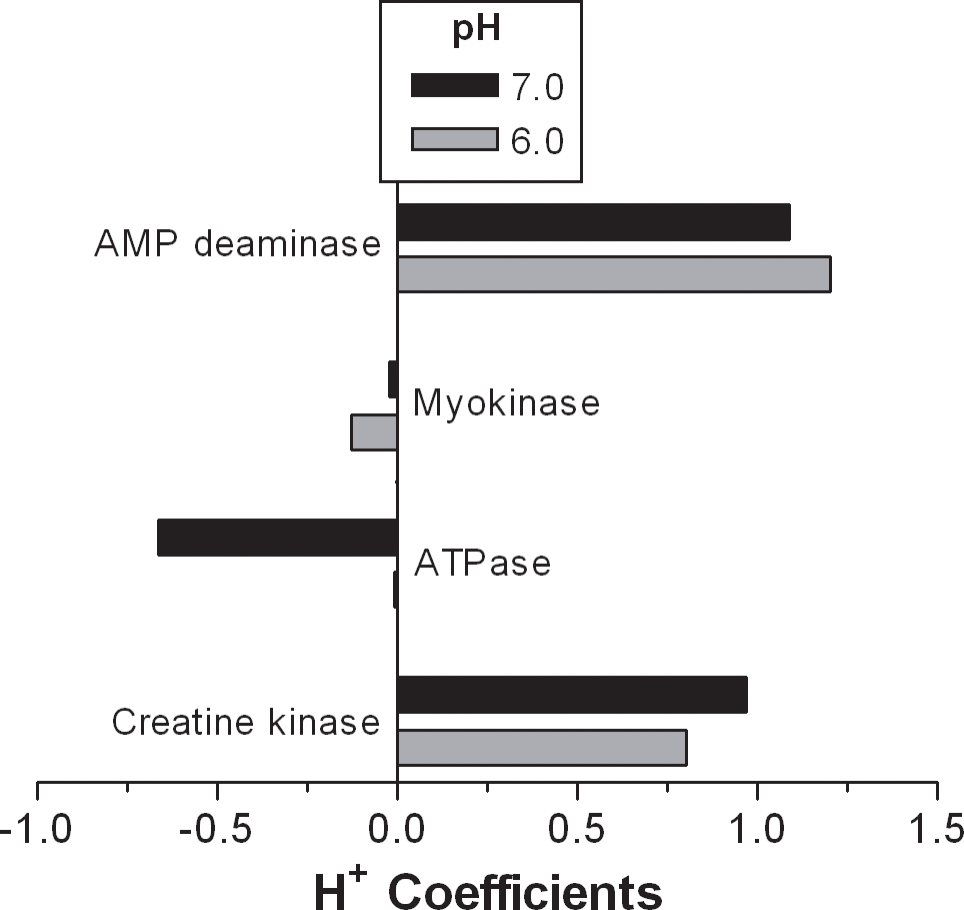
The H^+^ coefficients for reactions of the phosphagen energy system for cellular pH conditions of 6.0 and 7.0. As this is not a pathway, or a closed (coupled) energy system, H^+^ coefficients cannot be summed to get a net H^+^ coefficient.–‘ve coefficients = H^+^ release.

The H^+^ coefficients for the reactions of the phosphagen system, and phase 1 and 2 of glycolysis are presented in Fig 2A–2C. The polynomial equations for computing the H^+^ coefficients of all reactions are provided in Table 5. Note the metabolic H^+^ buffering capacity of the CK and AMPD reactions, and the H^+^ releasing potential of the ATPase reaction. Fig 3 presents the magnitude and direction of the H^+^ coefficients for the 4 reactions of the phosphagen system for pH conditions of 6.0 and 7.0. This presentation clearly reveals the potential of the phosphagen system for metabolically buffering H^+^ release.

**Table 5:** Polynomial equations for the H^+^ coefficient of each reaction and metabolic pathway for the pH range 6.0 to 7.0.

Reaction	H^+^ Coefficients
Reaction	Polynomial*
***Phosphagen System***	
HCrP + ADP + H^+^ ↔ Cr + ATP	A = 9.071, B = -4.631, C = 0.8177, D = -0.04592
ADP + ADP ↔ ATP + AMP	A = -3.442, B = 0.9321, C = -0.06334
AMP + H^+^ ↔ IMP + NH_4_	A = -48.99, B = 22.59, C = -3.365, D = 0.1656
ATP + H_2_O ↔ ADP + Pi +H^+^	A = -69.15, B = 33.03, C = -5.165, D = 0.2635
***Glycogenolysis***	
Glycogen(n) + HP ↔ Glycogen(n-1) + G_1_P	A = -281.0, B = 182.7, C = -44.15, D = 4.702, E = -0.1861
G_1_P ↔ G_6_P	A = 27.5, B = -12.74, C = 1.942, D = -0.0979
***Glycolysis***	
Glucose + ATP ↔ G_6_P + ADP + H^+^	A = -30.46, B = 16.66, C = -2.911, D = 0.1621
G_6_P ↔ F_6_P	A = -10.6, B = 4.857, C = -0.734, D = 0.03667
F_6_P + ATP ↔ F_1,6_P + ADP + H^+^	A = -83.84, B = 40.75, C = -6.491, D = 0.3379
F_1,6_P ↔ DHP + G3P	A = -1.007, B = 0.2371, C = -0.01347
DHP ↔ G_3_P	A = -65.39, B = 29.43, C = -4.36, D = 0.2134
G_3_P + HPi + NAD+ ↔ 1,3BPG + NADH + H^+^	A = 121.9, B = -59.52, C = 9.431, D = -0.4902
1,3BPG + ADP ↔ 3PG + ATP	A = -30.75, B = 16.07, C = -2.782, D = 0.1573
3PG ↔ 2PG	A = 200.7, B = -129.0, C = 30.86, D = -3.257, E = 0.128
2PG + ADP ↔ PEP	A = 62.94, B = -27.95, C = 4.082, D = -0.1967
PEP + ADP + H+ ↔ Pyr + ATP	A = 7.903, B = -6.276, C = 1.334, D = -0.08293
***Lactate Production***	
Pyr + NADH + H^+^ ↔ La + NAD^+^	A = 5.093, B = -2.33, C = 0.4995, D = -0.04777, E = 0.001719
***Net Glycolysis***	
Glycogen ↔ DHP + G3P	A = -2.245, B = 4.296, C = -1.138, D = 0.07857
Glucose ↔ DHP + G3P	A = -71.4, B = 37.33, C = -6.303, D = 0.3421
DHP + G_3_P ↔ Pyruvate	A = 205.8, B = -103.4, C = 16.64, D = -0.8694
DHP + G_3_P ↔ Lactate	A = 209.9, B = -104.3, C = 16.77, D = -0.8755
Glycogen ↔ Pyruvate	A = 203.5, B = -99.12, C = 15.5, D = -0.7908
Glucose ↔ Pyruvate	A = 134.4, B = -66.09, C = 10.34, D = -0.5273
Glycogen ↔ Lactate	A = 207.6, B = -100, C = 15.63, D = -0.797
Glucose ↔ Lactate	A = 138.5, B = -66.99, C = 10.46, D = -0.5334

* H^+^ coefficient = A + Bx + Cx^2^ + Dx^3^ + Ex^4^; x = pH

For glycolysis (Fig 2B and 2C), the reactions of phase I are clearly net H^+^ releasing, while reactions of phase II remain net H^+^ releasing. Of particular importance is the H^+^ coefficient change for the G3PD reaction, which becomes increasingly negative towards a value of -1.5 as pH falls from 7.0 to 6.0. This is caused by the increased fraction of H_2_PO^-1^ as pH decreases, which is a substrate for this reaction, and thereby can potentially contribute (fractional pH dependent) 2 H^+^ to the net reaction H^+^ release of the G3PD reaction. Finally, note that H^+^ exchange occurs for reactions that do not have H^+^ traditionally expressed as a substrate or product in the reaction. This occurs due to differences in the pK_a_ and therefore extent of H^+^ binding or dissociation between substrates and products.

**Fig 4:**
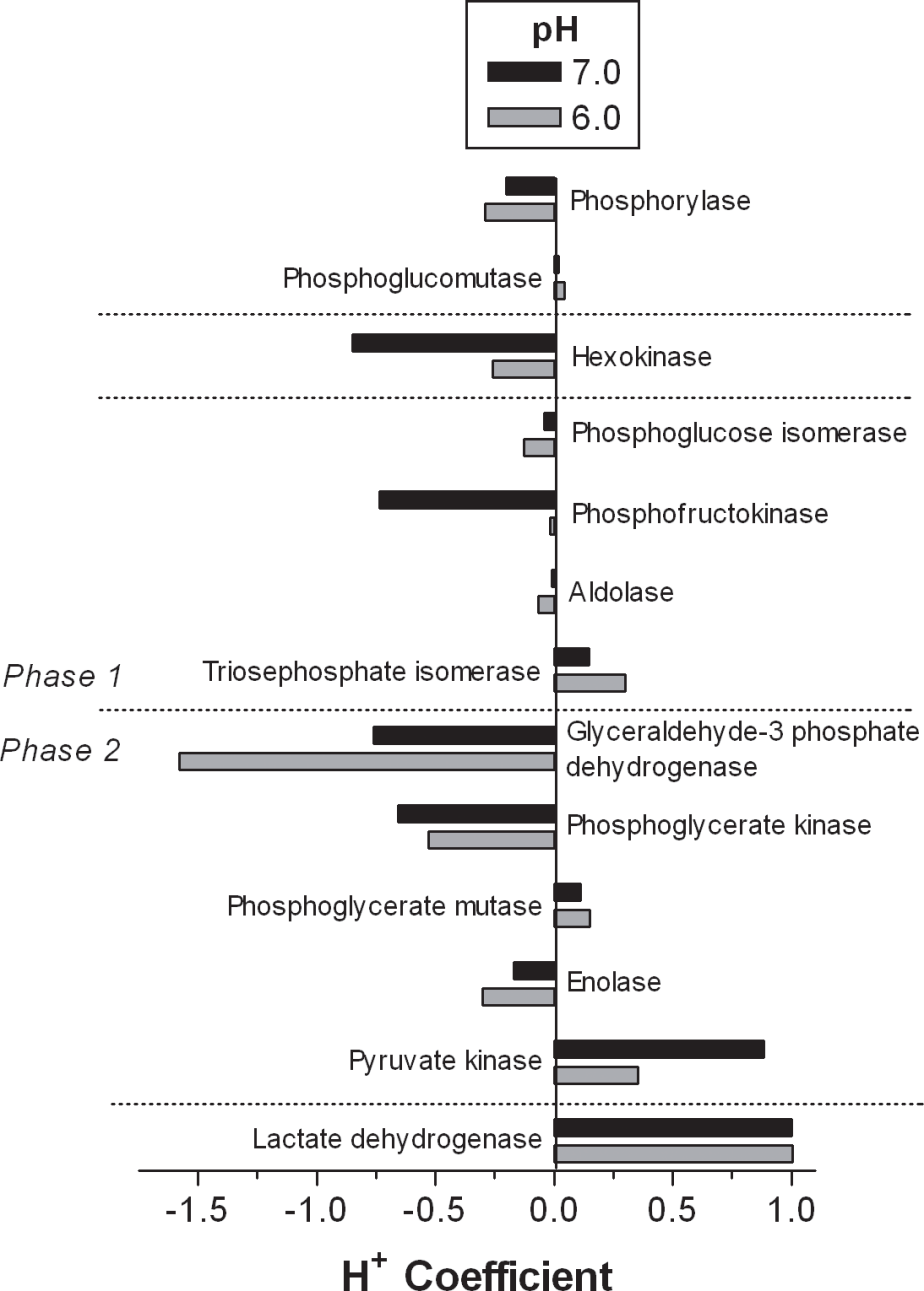
The H^+^ coefficients for reactions leading to glucose-6 phosphate production, the glycolytic pathway and lactate production for cellular pH conditions of 6.0 and 7.0.–‘ve coefficients = H^+^ release.

Fig 4 presents the magnitude and direction of the H^+^ coefficients for all reactions of glycogenolysis and glycolysis, including lactate production. As explained, the negative H^+^ coefficient of the glyceraldehyde-3-phosphate dehydrogenase reaction significantly contributes to the net acidifying potential of the glycolytic pathway, even when accounting for the H^+^ consumed in lactate production. This illustration also reveals that the incomplete flux of substrate through glycolysis, ending at the enolase reaction and preventing both the PK and LDH reactions, increases the H^+^ release of glycogen fueled glycolysis. Actual changes in the H^+^ coefficients would be from -2.01 to -3.78 at pH = 7.0, and from -3.97 to -4.67 at pH = 6.0.

**Fig 5:**
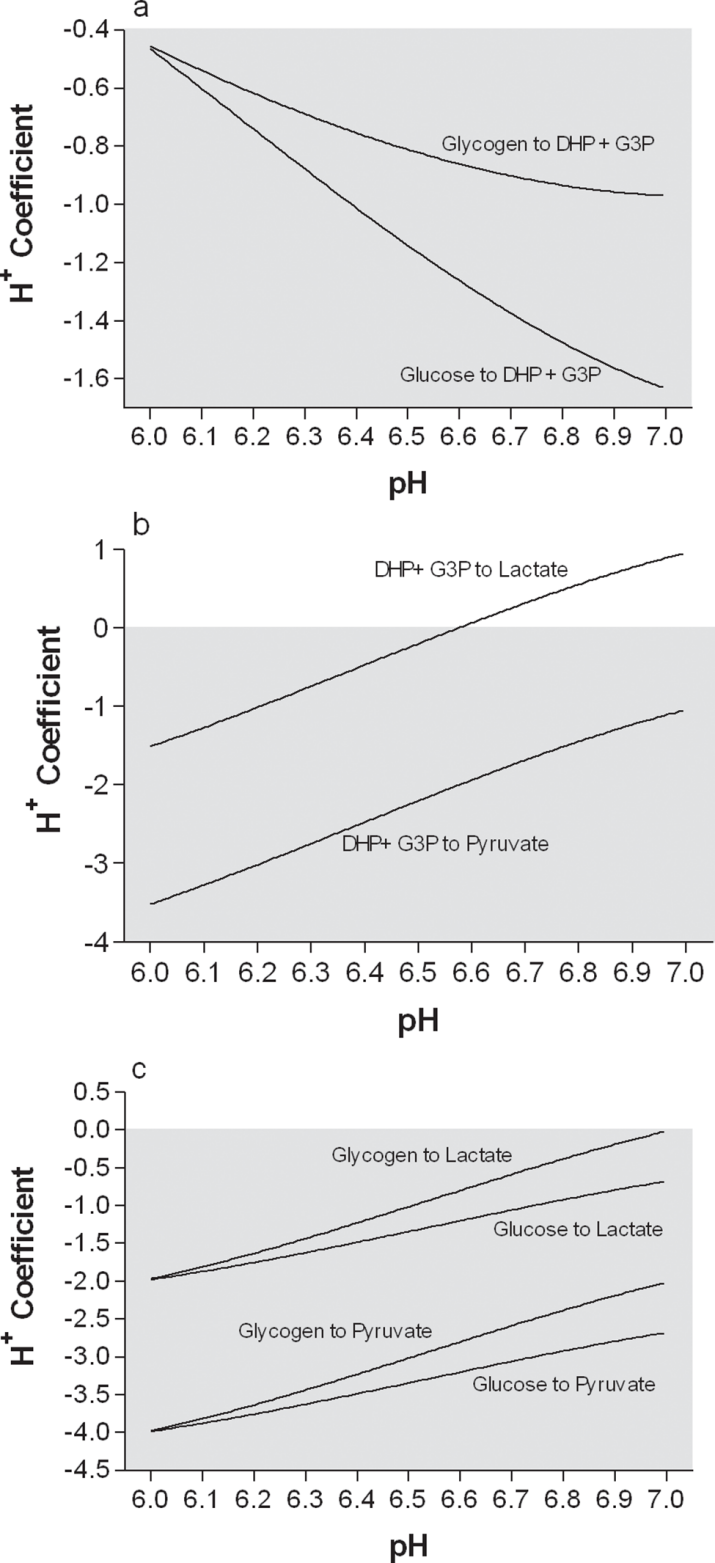
**Net H**^**+**^ **coefficients for the glycolytic pathway for a) phase 1, b) phase 2 ending in either pyruvate or lactate, and c) all of glycolysis starting with either glycogen or glucose, and ending in either pyruvate or lactate.–**‘ve coefficients (shaded area) = H^+^ release. Polynomial equations are presented in Table 5.

The H^+^ coefficients for phases 1 and 2 of glycolysis, and the total glycolytic pathway are presented in Fig 5A–5C. Data is presented for glycolysis starting with glycogen or glucose, as well as for ending with pyruvate or lactate. This presentation of the data clearly shows that as pH falls, glycolysis becomes more acidifying despite a near constant metabolic buffering potential of lactate production across the physiological pH range.

### Overview

Fractional binding data have been presented for the competitive binding between four cations (Mg^2+^, K^+^, Na^+^ and H^+^) to 21 metabolites, resulting in 104 different metabolite-cation complexes. Data of the fractional binding of H^+^ to these metabolite-cation complexes were applied to 17 reactions of skeletal muscle non-mitochondrial energy metabolism, and 8 conditions of the glycolytic pathway.

### Competitive cation binding

Data from Tables 1 and 4 reveal that adjusting H^+^ binding by competing cations appreciably changes the fraction of H^+^ bound metabolites across the physiological pH range. This is especially true for metabolites that competitively bind Mg^2+^, K^+^ and Na^+^, and the data are in agreement with the work of Kushmerick et al. [1] and Vinnakota et al. [2]. Discussion of these metabolites and related reactions will be structured by metabolic energy system. However, discussion of these findings relative to previous research is difficult for this manuscript is the first published compilation of the majority of data on fractional cation binding to all metabolites of non-mitochondrial energy metabolism, and the net H^+^ coefficients for all reactions of non-mitochondrial energy metabolism.

#### Phosphagen system metabolites and reactions

The phosphagen system comprises the metabolites ATP, ADP, AMP, IMP, NH_3_, Pi, CrP and Cr. The NIST database [13] presented incomplete data for these metabolites, with no acceptable data for any CrP-cation complex or the amine group proton (pK_a_ = 14.3) of IMP. Kushmerick et al. [1] presented data of fractional cation (Mg^2+^ and K^+^) binding for CrP, Pi, ADP and ATP, with data summed for cation free and H^+^ bound fractions of each metabolite. As Kushmerick et al. [1] did not present data solely for H^+^ bound fractions of each metabolite, it is difficult to interpet and compare the data of Kushmerick to these results. This is especially true for ADP, where the cation free complex varies from 17 to 27% of the total from pH 6.0 to 7.0. Nevertheless, for comparison of the present H^+^ coefficient data for the creatine kinase reaction from Fig 1 to the data from Kushmerick et al. [1] (their Fig 6c, p.C1743), the data are almost identical when substracting the cation free complex fractions. The same is true for comparison of the creatine kinase reaction H^+^ coefficient results to the data of Vinnakota et al. [2]. Such similar results across different investigations present important external validation to all of the phosphagen system metabolite and reaction computations of this investigation.

No prior published data exists for competitive cation binding of H^+^, Mg^2+^, K^+^ and Na^+^ to AMP, IMP and NH_3_. Interestingly, the AMPD reaction adds to the H^+^ uptake capacity of the CK reaction because of the high pK_a_ (9.26) of NH_3_-H. Consequently, the production of NH_3_ results in an immediate and near complete (fraction = 0.99945) consumption of a H^+^ to form the ammonium ion (NH_4_^+^). However, as AMPD is activated by acidosis, and that the capacity of this reaction is relatively small in skeletal muscle at < 20% of the capacity of the CK reaction, this reaction would do little to retard a developing acidosis in skeletal muscle.

Some researchers have assumed that the ATP hydrolysis of muscle contraction and the CK reaction are coupled, with the coupled reaction referred to as the Lohman reaction [1,4,6–8]. The initial alkalization of contracting skeletal muscle has been used as evidence for this interpretation [4,6]. However, the Lohman reaction is an error and oversimplification of the phosphagen energy system. Total muscle ATP turnover far exceeds the ATP turnover contribution from the creatine kinase reaction due to added ATP contributions from the adenylate kinase reaction and glycolysis. The near immediate stimulation of increased glycolytic flux during muscle contraction [15–17] prevents almost any muscle contraction condition from purely being fueled from CrP derived ATP. For quantitative proof of this fact Spriet et al. [14–16], Medbo et al. [18] and Bangsbo et al. [19] have presented evidence for total muscle ATP turnover during anoxic conditions to approximate 90 mmol/kg wet wt. over 3 min of intense exercise to contractile failure. The ATP capacity of the creatine kinase reaction approximates 30 mmol/kg wet wt [4,6,20,21], leaving a difference of 60 mmol/kg wet wt of ATP regeneration from the sum of the adenylate kinase reaction and glycolysis. During 3 min of sustained intense exercise, muscle CrP stores fall in an exponential decay function, but the half-life (t_0.5_) of CrP during such conditions is still in the order of 1 min (6). More recent data applying advanced ^31^P NMR equipment and methodologies during intense exercise [20] and in recovery from intense exercise [20], further supports this evidence. The data reveal that there is near instantaneous support of CrP derived ATP from the myokinase reaction and glycolysis. As such, there is no biochemical or research-based empirical evidence for the coupling of the CK and ATPase reactions (Lohman reaction) in contracting skeletal muscle.

#### Glycogenolysis and glycolysis metabolites and reactions

To the author’s knowledge, this is the first presentation of competitive cation binding computations to all metabolites and reactions of glycogenolysis and glycolysis. Vinnakota et al. [2] performed these computations and analyses for glycolysis, but did not present any of their data for specific metabolites or all reactions. However, the current H^+^ coefficient data for two of the reactions of glycolysis (G3PD, PGK) and the LDH reaction can be compared to Vinnakota et al. [2]. As for the data from the metabolites and reactions of the phosphagen system, the pH dependence of the H^+^ coefficients for these reactions are almost identical for this investigation and that of Vinnakota et al. [2]. This is important validation for the G3PD reaction, as the large H^+^ release of this reaction, with added H^+^ release during increasing acidosis, is an important feature of glycolysis. The data of this investigation and that of Vinnakota et al. [2] show that the G3PD reaction is the most acidifying reaction of glycolysis, and as such is important in accounting for the higher than typically interpreted H^+^ release assumption (2 H^+^ release per glucose flux) of the glycolytic pathway. This investigation shows that the net H^+^ release of glycolysis at pH 7.0 is 2 from glycogen, but 2.67 from glucose (Fig 5C). At pH 6.0, such numbers increase to 3.98 and 3.97, respectively. Consequently, glycolysis can be as much as 200% more acidifying than previously assumed, depending on cellular pH and the source of glucose.

As shown in Fig 2B and 2C, and Fig 4, some of the reactions of glycolysis that do not involve a H^+^ as a substrate or a product also can release H^+^ into solution. This occurs due to the products having less H^+^ binding than the substrates. For substrates that have less fractional H^+^ binding than products, there is net H^+^ consumption. The best examples of H^+^ release are the metabolites of the phosphorylase and enolase reactions. For the phosphorylase reaction, the pK_a_ values of G1P-H and HPO_3_-H are 6.09 and 6.75, respectively. While two H^+^ from H_2_PO_3_^-1^ are liberated, one is used to complete a hydroxyl group on carbon-4 of the remaining terminal glucose residue of glycogen. The remaining H^+^ activity is dictated by the pK_a_. The lower fractional protonation of G1P than Pi at any pH causes a net H^+^ release during the reaction. Such H^+^ release varies from 0.2 to 0.29 from pH 7.0 to 6.0, respectively.

The same principles apply to the enolase reaction, where the pK_a_ values of 2PG-H and PEP-H are 7.0 and 6.35, respectively. Across the physiological pH range, there is greater fractional protonation of 2PG than PEP, causing net H^+^ release during the reaction. Such fractional H^+^ release varies from 0.17 to 0.3 from pH 7.0 to 6.0, respectively.

An example reaction involving net H^+^ consumption is the TPI reaction, where the pK_a_ values of DHAP-H and G3P-H are 5.90 and 6.45, respectively. Here, the product G3P has greater fractional protonation than the substrate DHAP, causing H^+^ uptake from solution. Such fractional H^+^ uptake varies from 0.15 to 0.3 from pH 7.0 to 6.0, respectively.

#### Glycolysis ending in lactate

The H^+^ consumption of lactate production is largely pH independent, varying little from 1.0004 to 1.004 from pH 7.0 to 6.0. Thus, production of two lactate results in the consumption of 2 H^+^, regardless of pH across the physiological range.

Traditionally, the H^+^ yield of glycolysis has been assumed balanced by the H^+^ consumption of lactate production [5–12]. However, the H^+^ coefficient of glycolysis is only close to 2.0 at pH 7.0, and only when glycolysis is fueled by glycogen and not glucose. As pH falls, the greater H^+^ release from glycolysis exceeds the H^+^ uptake from lactate as soon as pH falls below 7.0 (Fig 5C). When commencing glycolysis from glucose, there is always net H^+^ release despite lactate production. Based on this data, and due to the mix of blood glucose and glycogen-derived glucose during exercise, it is reasonable to view glycolysis in contracting skeletal muscle as always causing net H^+^ release, with such release increasing with continuing acidosis or sustained glycolytic flux with constrained lactate production. The stoichiometry between glycolytic flux and H^+^ release is therefore summarized as ranging from 0.0 to 4.0 depending on lactate production (which decreases net glycolytic H^+^ release), cellular pH and the source of glucose substrate.

### Applications of findings

The academic and research inquiry topics of the metabolic biochemistry of contracting skeletal muscle, as well as the buffer capacity of skeletal muscle, can benefit from this research. For the first time, comprehensive data is presented allowing computations of the fraction of H^+^ binding for all metabolites and reactions of non-mitochondrial energy metabolism.

Such data has computed the actual H^+^ balance of key reactions and the glycolytic pathway, which for the first time, has been revealed to be always acidifying to skeletal muscle and with up to twice the H^+^ release as previously assumed. Academics and reach scientists alike are now equipped with data to more validly profile the balance of H^+^ during intermediary metabolism, and thereby allow more valid biochemical understanding of the cause of metabolic acidosis, and the components of metabolic H^+^ buffering.

### Limitations

This data is by no means final. Of the 17 metabolites studied in this investigation, incomplete data for competitive cation binding occurred for 6 (29%), and it remains unclear how many of the remaining metabolites that had data were also incomplete in cation binding representation. As shown in Table 3, the fraction of H^+^ binding can be lowered when accounting for competitive cation binding in computations. This may be meaningful for the glycolytic intermediates that currently have no data of the influence of the competing cations Mg^2+^, K^+^ and Na^+^ to fractional H^+^ binding (G6P, F6P, G3P, 1,3BPG, 3PG, 2PG). It is possible that future progress with research that quantifies the K_M+_ data for cations and glycolytic intermediates will result in slight reductions in the H^+^ release for glycolysis. However, until this happens, the present data are the most valid that exist.

It is also important to understand that the computations for metabolic pathways such as glycolysis ending in pyruvate vs. lactate, assume complete flux of metabolic intermediates through the pathway (e.g. to pyruvate and lactate, respectively). Any accumulation of intermediates within the pathway, which does happen in-vivo in contracting skeletal muscle, would require adjustment to the H^+^ stoichiometry presented for specific pathways.

## Supporting information

S1 File An Excel spreadsheet file containing all data used to compute metabolite and reaction proton (H^+^) exchange. (XLS)

S2 File A Prism graphics and curve fitting file containing data for both metabolites and reactions of the phosphagen and glycolytic energy systems. (PZF)

S3 File A Prism graphics and curve fitting file containing data for metabolites of the phosphagen and glycolytic energy systems. (PZM)
